# The Difficulties of the Schools

**Published:** 1917-09-08

**Authors:** 


### MEDICAL EDUCATION IN WARTIME.

### The Difficulties of the Schools.

The situation in most of our large teaching hospitals at the outbreak of hostilities was one of great ambiguity as regards both the staff and the students.

The rival claims of devotion to country and loyalty to the *alma mater*, which, whatever might be the urgency of international affairs, was bound to carry on the work for which it was founded, gave rise to many hot discussions and many secret heartburnings. Many students were, of course, already enrolled in the crack territorial regiments, and were thus naturally mobilised on the declaration of war, irrespective of the fact that their studies were in some cases nearing completion.

But by far the larger number were in a position to decide which course they should take, and many and various were the *pros* and *cons* urged for each alternative. Those far-seeing ones who believed in a war of long duration naturally elected to stay behind and complete their course, holding, quite rightly, that as medical men they would be of greater service to the national cause than if they joined in a combatant capacity. But a considerable number felt it to be a duty to take arms in the defence of their country in her hour of need, believing that the struggle would be of so short and decisive a character that to delay would be to lose the opportunity of playing a man's part in the struggle. Yet a third alternative shortly presented itself in the rapid organisation of the many relief units which, under the *ægis* of the two Red Cross Societies, were, as we know, dispatched to the scene of hostilities with such admirable promptitude. But on the whole it may be said that in the early months most of the hospitals presented an appearance which, to the casual eye at all events, did not differ very greatly from the normal. A few of the staff had left for war service at home or abroad; an increased activity was noticeable among those whose qualifying examinations were imminent; and in some hospitals batches of cadets from the universities were being put through a rapid course of first-aid work, with the object of rendering themselves immediately useful if required. Many students enrolled themselves in the ambulance section of the O.T.C., in which a vigorous system of training at week-end camps, daily drills, and lectures had been organised at the different headquarters. The Navy, too, claimed its quota, for the Admiralty authorities, realising that fourth and fifth year students could be utilised in many capacities where it would be hardly justifiable to retain the services of a fully-qualified man, instituted the rank of surgeon-probationer. Such men were and are employed in the main on smaller craft, minesweepers, destroyers, sloops, and the like, and are released after a certain period of service to complete their studies, re-entering the Navy, when qualified, as full surgeons.

Such, in brief, was the situation which obtained during 1914 and the earlier months of 1915.

But as the demands of the newly organised armies for medical officers became more insistent, and the recruiting authorities enrolled more and more young men, the governing bodies of all the teaching hospitals were faced with a two-fold problem—how to keep up the standard of teaching in the scientific portion of the curriculum when students and fees were growing scarcer, and how to fill their house appointments when every newly qualified man was needed in the Army Medical Corps. The position of the student, for example, who had passed his first qualifying examination and spent six months or so in the dissecting-room was by no means clear. Was he justified in remaining at his studies in view of the probably lengthy duration of the war, or, in doing so, did he incur the stigma of shirking, of consulting his own interests rather than those of his country? The War Office authorities were appealed to, but without success. Letters appeared in the press from the heads of the profession, each urging a different course, some

**MEDICAL EDUCATION IN WARTIME**—(*concluded*).

vituperating the students who stayed on, others telling them they were fulfilling their national obligations in qualifying as soon as possible. The effect of this diversity of opinion was unfortunate. Lacking official ratification of their position, many keen men threw over their work and joined up, the rest continued in a rather shamefaced fashion, walking in fear of recruiting sergeants and ladies with white flags.

Late in the summer of 1915 the edict came forth that fourth and fifth year men, and all those who would reach this position within the next few months, were on no account to leave their work, and that the rest should join the Army forthwith. Meanwhile, the question of obtaining qualified house officers, especially in the smaller hospitals, was growing more and more acute. In normal times a hospital with a large school can only grant the position of house surgeon or physician to about a third of the men qualifying at any given time. In those with fewer students supply and demand are fairly evenly balanced, and it was in these that the pinch began first to make itself felt. The columns of The Hospital have from time to time furnished a fairly complete record of the various expedients adopted—reduction of the number of men appointed with a corresponding increase in their duties; abolition of the posts of registrar and resident surgical officer, these places being taken by men on the permanent staff for short periods at a time; and the opening of many vacancies to senior students under qualified supervision. The hospitals had to learn the same lesson of "carry on" that was exercising the rest of the civilian population.

There was, however, one exception to the general short-handedness—namely, those hospitals which admitted women students, and in which medical women were allowed to hold qualified posts. Early in the war a vigorous newspaper campaign was organised to popularise the profession as a means of livelihood for women, and the sterling work done by the numerous hospitals and ambulances staffed and managed entirely by lady doctors did much to silence any opposition which such advocacy might otherwise have met. There would be, it was urged, a shortage of medical men for some years after the war; the normal supply of those commencing study had been cut off; and not a few who had already begun their studies and left them for service would, in all probability, never return from the field of battle. In the meantime the needs of the civil population were growing greater rather than less; and it was seen that if we were to maintain our position as a nation the life of every infant must be zealously safeguarded. Thus advocated, it is hardly surprising that there was a large increase in the number of entries at the hospitals taking lady students. In London, however, there was of these but one—namely, the Royal Free Hospital. In view, however, of the anticipated influx, several other hospitals decided to open their doors to women. The movement is, as yet, of too recent date for there to be any appreciable increase in the number of lady doctors. Yet we cannot but think that any wholesale booming of an already overcrowded profession is against the best interests of medical practitioners generally. The training is a lengthy one, and the war, with the abnormal conditions it has produced, will, we trust, be a thing of the past ere any of these new recruits find their place upon the market.

Another notable if a minor feature is the return to the curriculum of men who, after passing one or two of the qualifying examinations, had left medicine for some other walk in life. In one case a young and highly successful artist, whose canvases have been prominently displayed at the New English Art Club, returned, and completed in record time a medical curriculum that he had commenced years before. A prosperous dentist, also, returned to complete the degree in medicine that he had discarded for dentistry in his youth. No doubt many such instances could be recorded.

The advent of conscription, with its manifold problems of exemption, naturally affected the student community more than most. The position of the fourth and fifth years was assured. But again the third-year man claimed attention. On the whole the Tribunals were disposed to take a lenient view. Students preparing for their intermediate examination were, on the representations of the Dean of their school, granted three months' exemption in which to pass, with a broad hint that a further time limit would be fixed in the event of failure. Absolute uniformity was not obtainable, but in general the Tribunals held that medical students were fulfilling their duty in remaining behind. That the Army authorities also were of this opinion was shown by the number of students who, though serving in combatant corps, were released to resume their interrupted studies. The question of calling up newly qualified men had also to be considered. At first the authorities were disposed to allow every hospital its customary quota of house officers, each man being granted three months' further exemption from the Army on this ground. But soon a drastic policy of cutting down was introduced. In one large hospital where usually there are twenty house men, only eight were allowed, and all the minor posts filled by men who have completed their house appointments and are awaiting a vacancy upon the staff were abolished. The later Order of 1917, calling up all medical men of military age, affected not only the younger men but also those holding permanent posts on the staff. The problem of carrying on, of providing adequate treatment for the poor, and sufficient teaching for the dwindling body of students, is one that is taxing the Deans of Medical Faculties to the utmost. Unrivalled opportunities are available for keen senior students to obtain experience of a kind that many a qualified house officer in former days would have jumped at. On the whole the future prospects for medical education, whilst not exactly bright, are redeemed by the splendid spirit shown by all concerned in making the best of curtailed resources and diminished opportunities. One thing is essential, that by national support or, as hitherto, by purely voluntary effort, the splendid tradition of medical teaching in this country must not be permitted to decline from its high position in public esteem.

